# Supplementary material for: Evaluation of the Roche SARS-CoV-2 Rapid Antibody Test in Samples from Vaccinated Individuals
Source: Microbiol Spectr. 2022 May 16;10(3):e02709-21. doi: 10.1128/spectrum.02709-21 (PMC9241600; doi:10.1128/spectrum.02709-21)
Supplement: SUPPLEMENTAL FILE 1 — Supplemental material. Download spectrum.02709-21-s001.pdf, PDF file, 0.3 MB [file spectrum.02709-21-s001.pdf]

Supplemental

Supplemental Table 1: Boca Biolistics sample panel

<table>
<tr><th></th><th>Sampled prior to vaccination</th><th>Sampled after first dose</th><th>Sampled ≥ 14 days after second dose</th><th>Sub-total</th></tr>
<tr><td>Moderna</td><td rowspan="3">8*</td><td>5[†]</td><td>19</td><td>24</td></tr>
<tr><td>Pfizer-BioNTech</td><td>8</td><td>34</td><td>42</td></tr>
<tr><td>Sub-total</td><td>13</td><td>53</td><td>66</td></tr>
<tr><td>Total samples</td><td colspan="3">74</td><td></td></tr>
</table>

*2 samples came from donors who were previously diagnosed with COVID-19 (known infection, unknown whether diagnosis was PCR-confirmed). Diagnosis was 3 months prior to the date of the first draw for one individual and 6 months for the second individual

[†]Duplicate samples from the same donor, taken at the same visit, were available – only the first sample was used in analyses

Supplemental Table 2: Interpretation of SARS-CoV-2 Rapid Antibody Test for semi-quantitative analysis

Result	Symbol*	Description
**IgM positive**	((+)), (+), +, ++, +++	• Control line and IgM signal line both visible<br>• Faint signal lines are rated as positive<br>• Signal intensity will be assessed using the color scale
**IgG positive**	((+)), (+), +, ++, +++	• Control line and IgG signal line both visible<br>• Faint signal lines are rated as positive<br>• Signal intensity will be assessed using the color scale
**Negative**	-	• Control line visible<br>• No signal line
**Invalid**	/	• No control line<br>• Unusual background of the test strip<br>• Further reasons

*Daylight lamps were used to assist the visual interpretation of the bands.

Results from the SARS-CoV-2 Rapid Antibody Test were classified as invalid, negative or one of the five levels of increasing positivity based upon line intensity (((+)), (+), +, ++ or +++)

Supplemental Table 3: Agreement between SARS-CoV-2 Rapid Antibody Test (IgG and IgM) and Elecsys Anti-SARS-CoV-2 S assay (reference test) qualitative measurements, as measured by lot and evaluator for in-house negative panel

		**Evaluator 1**		**Evaluator 2**	
		**Lot 1**	**Lot 2**	**Lot 1**	**Lot 2**
**IgG**	**N**	15	15	15	15
	**N -**	15	15	15	15
	**FP**	0	0	0	0
	**TN**	15	15	15	15
	NPA, %	*100*	*100*	*100*	*100*
	lower CI	*78.2*	*78.2*	*78.2*	*78.2*
	upper CI	*100*	*100*	*100*	*100*
**IgM**	**N**	15	15	15	15
	**N -**	15	15	15	15
	**FP**	0	0	0	0
	**TN**	15	15	15	15
	NPA, %	*100*	*100*	*100*	*100*
	lower CI	*78.2*	*78.2*	*78.2*	*78.2*
	upper CI	*100*	*100*	*100*	*100*

-, negative by the reference test (Elecsys Anti-SARS-CoV-2 S assay); CI, 95% confidence intervals; FP, false-positive; TN, true-negative; NPA, negative percent agreement

Supplemental Table 4. Kendall's correlation between the SARS-CoV-2 Rapid Antibody Test semi-quantitative results and the Elecsys Anti-SARS-CoV-2 S total antibody titer for samples from individuals vaccinated with Moderna mRNA-1273 or Pfizer-BioNTech BNT162b2 (the analysis includes only those who have had two doses). Moderna: n=19 for each lot/evaluator. Pfizer: n=34 for each lot/evaluator. Significant p-values indicated in red.

		**Kendall's τ**	**p-value**
**mRNA-1273**			
**IgM**			
Elecsys_Quant	Rapid AB test, Lot 1 Evaluator 1	-0.0875	0.6541
Elecsys_Quant	Rapid AB test, Lot 1 Evaluator 2	0.1101	0.5722
Elecsys_Quant	Rapid AB test, Lot 2 Evaluator 1	-0.0875	0.6541
Elecsys_Quant	Rapid AB test, Lot 2 Evaluator 2	0.0097	0.9603
**IgG**			
Elecsys_Quant	Rapid AB test, Lot 1 Evaluator 1	0.5729	0.0029
Elecsys_Quant	Rapid AB test, Lot 1 Evaluator 2	0.3712	0.0479
Elecsys_Quant	Rapid AB test, Lot 2 Evaluator 1	0.599	0.0019
Elecsys_Quant	Rapid AB test, Lot 2 Evaluator 2	0.3009	0.1103
**BNT162b2**			
**IgM**			
Elecsys_Quant	Rapid AB test, Lot 1 Evaluator 1	0.1126	0.4304
Elecsys_Quant	Rapid AB test, Lot 1 Evaluator 2	0.1977	0.1667
Elecsys_Quant	Rapid AB test, Lot 2 Evaluator 1	0.1021	0.4718
Elecsys_Quant	Rapid AB test, Lot 2 Evaluator 2	0.0933	0.5136

**IgG**			
Elecsys_Quant	Rapid AB test, Lot 1 Evaluator 1	0.6833	<0.0001
Elecsys_Quant	Rapid AB test, Lot 1 Evaluator 2	0.4075	0.0035
Elecsys_Quant	Rapid AB test, Lot 2 Evaluator 1	0.5881	<0.0001
Elecsys_Quant	Rapid AB test, Lot 2 Evaluator 2	0.5956	<0.0001

Elecsys, Elecsys Anti-SARS-CoV-2 S assay; Quant, quantitative; Rapid AB test, SARS-CoV-2 Rapid Antibody Test

Supplemental Figure 1: Forest plot showing accuracy for SARS-CoV-2 Rapid Antibody Test (IgG), lot-to-lot and evaluator-to-evaluator, after vaccination with Moderna mRNA-1273 or Pfizer-BioNTech BNT162b2.

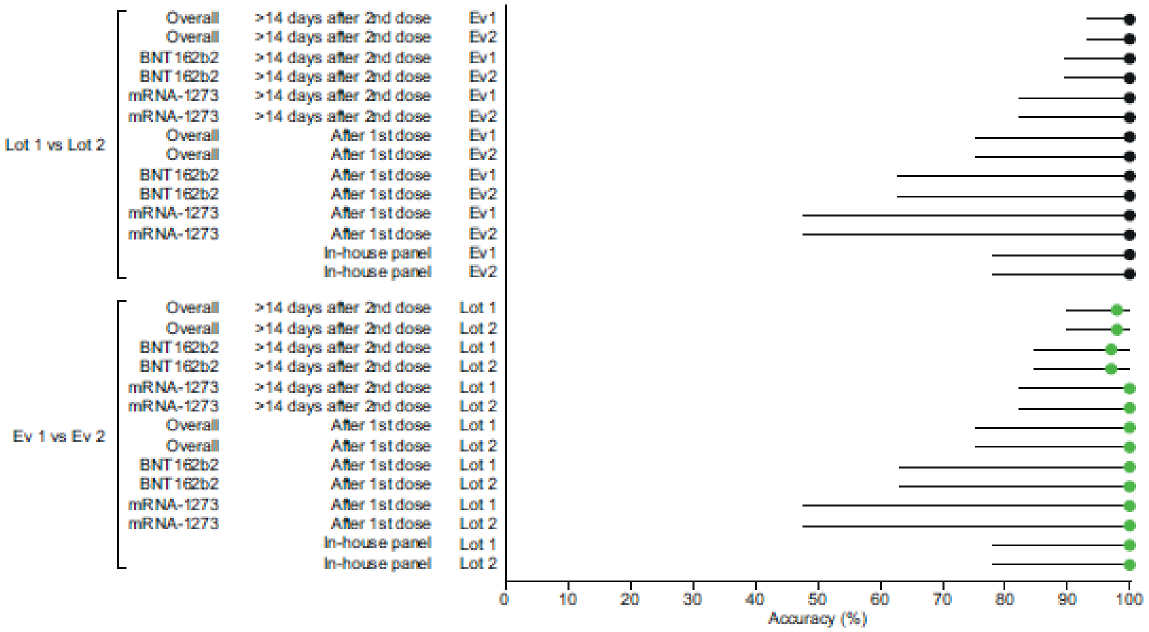

The horizontal line represents the 95% confidence intervals of the point estimate.

Overall represents the combined accuracy data for mRNA-1273 and BNT162b2

Ev, evaluator

Supplemental Figure 2: Forest plot showing accuracy estimates for SARS-CoV-2 Rapid Antibody Test (IgM), lot-to-lot and evaluator-to-evaluator, after vaccination with Moderna mRNA-1273 of Pfizer-BioNTech BNT162b2.

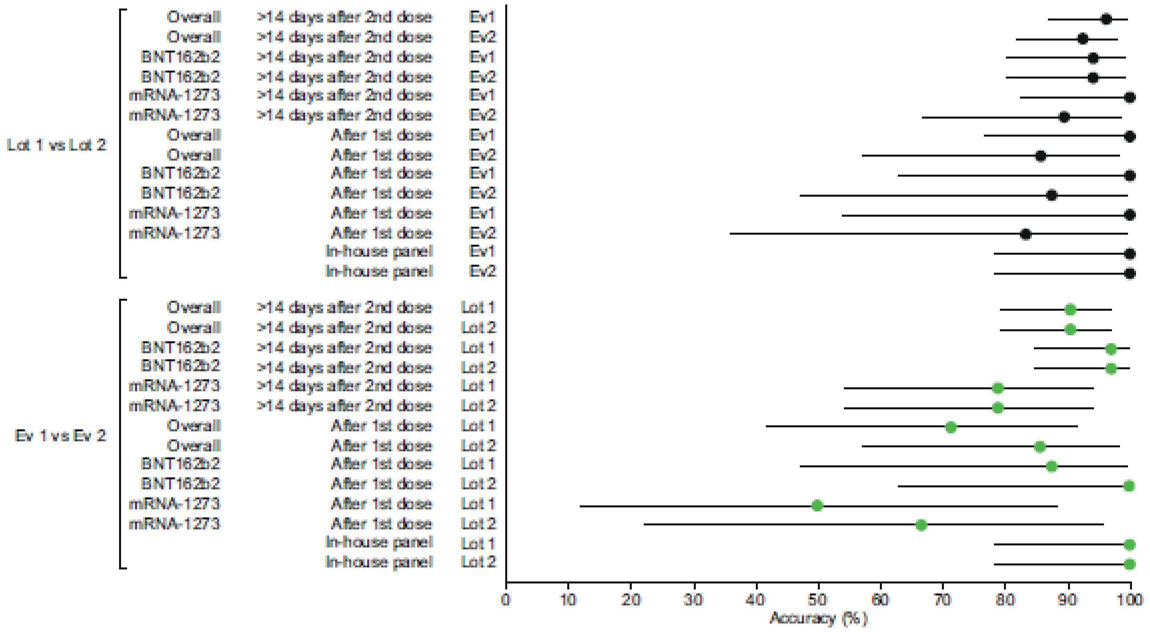

The horizontal line represents the 95% confidence intervals of the point estimate.
Overall represents the combined accuracy data for mRNA-1273 and BNT162b2

Ev, evaluator

Supplemental Figure 3: Longitudinal analysis of Elecsys Anti-SARS-CoV-2 S assay total antibody titers in individuals with serial samples available after vaccination with Pfizer-BioNTech BNT162b2

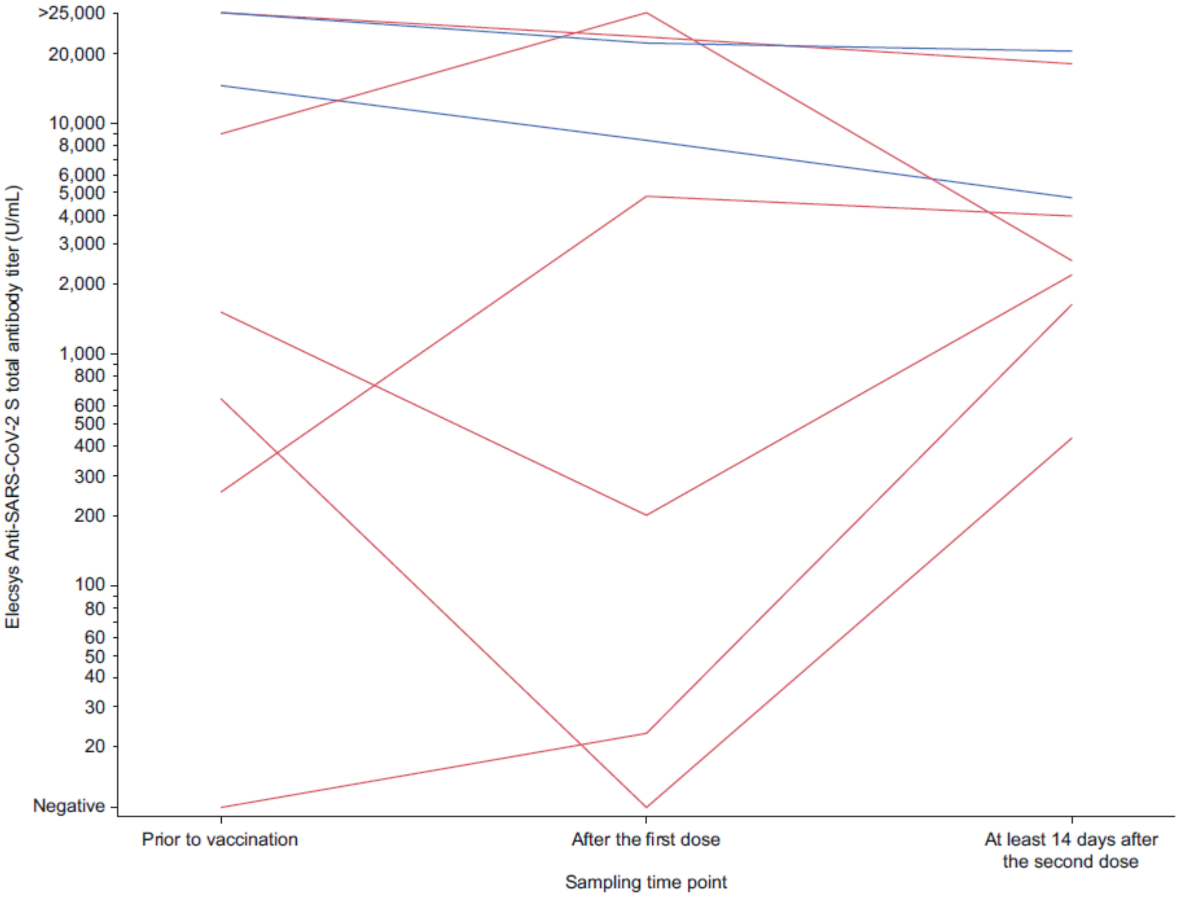

Blue color represents those individuals diagnosed with COVID-19 prior to vaccination

Section & Topic	No	Item	Reported on page #
**TITLE OR ABSTRACT**			
	**1**	Identification as a study of diagnostic accuracy using at least one measure of accuracy (such as sensitivity, specificity, predictive values, or AUC)	2 (agreement)
**ABSTRACT**			
	**2**	Structured summary of study design, methods, results, and conclusions (for specific guidance, see STARD for Abstracts)	1/2
**INTRODUCTION**			
	**3**	Scientific and clinical background, including the intended use and clinical role of the index test	4/5
	**4**	Study objectives and hypotheses	5
**METHODS**			
Study design	**5**	Whether data collection was planned before the index test and reference standard were performed (prospective study) or after (retrospective study)	5
Participants	**6**	Eligibility criteria	6
	**7**	On what basis potentially eligible participants were identified (such as symptoms, results from previous tests, inclusion in registry)	6
	**8**	Where and when potentially eligible participants were identified (setting, location and dates)	6
	**9**	Whether participants formed a consecutive, random or convenience series	NA
Test methods	**10a**	Index test, in sufficient detail to allow replication	7 + Supplemental Table 2
	**10b**	Reference standard, in sufficient detail to allow replication	7
	**11**	Rationale for choosing the reference standard (if alternatives exist)	NA
	**12a**	Definition of and rationale for test positivity cut-offs or result categories of the index test, distinguishing pre-specified from exploratory	7
	**12b**	Definition of and rationale for test positivity cut-offs or result categories of the reference standard, distinguishing pre-specified from exploratory	7
	**13a**	Whether clinical information and reference standard results were available to the performers/readers of the index test	6/8
	**13b**	Whether clinical information and index test results were available to the assessors of the reference standard	6/8
Analysis	**14**	Methods for estimating or comparing measures of diagnostic accuracy	8
	**15**	How indeterminate index test or reference standard results were handled	7
	**16**	How missing data on the index test and reference standard were handled	7
	**17**	Any analyses of variability in diagnostic accuracy, distinguishing pre-specified from exploratory	8
	**18**	Intended sample size and how it was determined	6
**RESULTS**			
Participants	**19**	Flow of participants, using a diagram	NA

	**20**	Baseline demographic and clinical characteristics of participants	8 + 26/27 (Table 1)
	**21a**	Distribution of severity of disease in those with the target condition	NA
	**21b**	Distribution of alternative diagnoses in those without the target condition	NA
	**22**	Time interval and any clinical interventions between index test and reference standard	NA
Test results	**23**	Cross tabulation of the index test results (or their distribution) by the results of the reference standard	28/29 (Table 2)
	**24**	Estimates of diagnostic accuracy and their precision (such as 95% confidence intervals)	28/29 (Table 2)
	**25**	Any adverse events from performing the index test or the reference standard	NA
**DISCUSSION**			
	**26**	Study limitations, including sources of potential bias, statistical uncertainty, and generalisability	14
	**27**	Implications for practice, including the intended use and clinical role of the index test	14/15
**OTHER INFORMATION**			
	**28**	Registration number and name of registry	NA
	**29**	Where the full study protocol can be accessed	NA
	**30**	Sources of funding and other support; role of funders	NA

61
